# Volumetric brain reductions in adult patients with phenylketonuria and their relationship with blood phenylalanine levels

**DOI:** 10.1186/s11689-024-09553-w

**Published:** 2024-06-21

**Authors:** Jèssica Pardo, Clara Capdevila-Lacasa, Bàrbara Segura, Adriana Pané, Cristina Montserrat, Maria de Talló Forga-Visa, Pedro J. Moreno, Glòria Garrabou, Josep M. Grau-Junyent, Carme Junqué, Consortium PKU.cat., Ana Argudo-Ramírez, Blanca Barrau-Martínez, Judith Cantó, Jaume Campistol, Francesc Cardellach, Climent Casals-Pascual, Gemma Chiva-Blanch, Dolores García-Arenas, Francesc Josep García-García, Judit García-Villoria, José Manuel González de Aledo-Castillo, Arnau González-Rodríguez, Mariona Guitart-Mampel, Paula Isern, Amanda Jiménez, Berta Laudo, Rafael Llorach, Félix Andújar-Sánchez, Rosa López-Galera, Silvia Mª Meavilla, José Cesar Milisenda, Blai Morales, Pedro Juan Moreno-Lozano, Julián Moreno, Mònica Nos, Aida Ormazabal, Montserrat Ortega Ferrer, Emilio Ortega, Joan Padrosa, Abraham José Paredes, Elisa Rubio, Ester Tobías, Josep Torremade, Mireia Urpi-Sarda, Laura Valls, Roser Ventura, Andrea Vergara-Gómez, Judith Viaplana, Clara Viñals

**Affiliations:** 1 Institute of Neurosciences, Medical Psychology Unit, Department of Medicine, University of Barcelona, C/ Casanova 143, Barcelona, Catalonia 08036 Spain; 2 Fundació de Recerca Clínic Barcelona-Institut d’Investigacions Biomèdiques August Pi I Sunyer (FRCB-IDIBAPS), Barcelona, Catalonia Spain; 3 Biomedical Research Networking Center On Neurodegenerative Diseases (CIBERNED: CB06/05/0018-ISCIII), Barcelona, Catalonia Spain; 4 Biomedical Research Networking Center on Physiopathology of Obesity and Nutrition (CIBEROBN), Barcelona, Catalonia Spain; 5 Endocrinology and Nutrition Department, Adult Inherited Metabolic Disorders Unit (UECMA), Hospital Clínic de Barcelona, Barcelona, Catalonia Spain; 6 Internal Medicine Department, Adult Inherited Metabolic Disorders Unit (UECMA), Hospital Clínic de Barcelona, Barcelona, Catalonia Spain; 7 Inherited Metabolic Diseases and Muscle Disorders Research, Centre de Recerca Biomèdica CELLEX – Institut d’Investigacions Biomèdiques August Pi i Sunyer (IDIBAPS) and Faculty of Medicine and Health Sciences, University of Barcelona, Barcelona, Catalonia Spain; 8 Biomedical Research Networking Center on Rare Diseases (CIBERER), Barcelona, Catalonia Spain

**Keywords:** Phenylketonuria, Adult early-treated patients, Blood phenylalanine levels, Neuroimaging, Volumetry, Neuropsychological assessment

## Abstract

**Background:**

Continued dietary treatment since early diagnosis through newborn screening programs usually prevents brain-related complications in phenylketonuria (PKU). However, subtle neurocognitive and brain alterations may be observed in some adult patients despite early treatment. Nevertheless, neuropsychological and neuroimaging studies in the field remain scarce.

**Objectives:**

This work aimed to determine possible neuropsychological and structural brain alterations in treated adult patients with PKU.

**Methods:**

Thirty-five patients with PKU and 22 healthy controls (HC) underwent neuropsychological assessment and T1-weighted magnetic resonance imaging on a 3 T scanner. *FreeSurfer* (v.7.1) was used to obtain volumetric measures and SPSS (v27.0.1.0) was used to analyze sociodemographic, neuropsychological, volumetric, and clinical data (*p* < 0.05).

**Results:**

Adult patients with PKU showed significantly lower performance than HC in Full Scale IQ (t = 2.67; *p* = .010) from the WAIS-IV. The PKU group also showed significantly lower volumes than HC in the pallidum (U = 224.000; *p* = .008), hippocampus (U = 243.000; *p* = .020), amygdala (U = 200.000; *p* = .002), and brainstem (t = 3.17; *p* = .006) as well as in total cerebral white matter volume (U = 175.000; *p* = .001). Blood phenylalanine (Phe) levels in PKU patients were negatively correlated with the pallidum (*r* = -0.417; *p* = .013) and brainstem (*r* = -0.455, *p* = .006) volumes.

**Conclusions:**

Adult patients with early-treated PKU showed significantly lower global intelligence than HC. Moreover, these patients showed reduced global white matter volume as well as reductions in the volume of several subcortical grey matter structures, which might be related to the existence of underlying neurodevelopmental alterations. Higher blood Phe levels were also negatively correlated with pallidum and brainstem, suggesting a higher vulnerability of these structures to Phe toxicity.

## Background

Phenylketonuria (PKU) (OMIM #261600) and its milder variant hyperphenylalaninemia (HPA) (OMIM #261630) are inborn errors of amino acid metabolism. These conditions result from homozygous or compound heterozygous mutations in the phenylalanine hydroxylase gene (OMIM #612349), which encodes an enzyme responsible for the conversion of phenylalanine (Phe) into tyrosine. The disruption of this enzymatic pathway leads to a notable accumulation of Phe and a corresponding decrease in tyrosine levels. This imbalance manifests in elevated Phe concentrations in the brain, inducing various neuropathological outcomes. Among these neurotoxic effects [1], there is a relevant effect on the dopaminergic system [2], along with an adverse impact on oligodendroglia, causing defective myelin synthesis [3, 4].

Blood Phe concentration is the primary reported marker of metabolic control. Although target ranges are based on plasma Phe (the upper target for adults has been set at 600 μmol/L according to the European guidelines) [1], patients are routinely monitored using dried bloodspot (DBS) specimens due to the convenience of their collection [5].

PKU early diagnosis through newborn screening (NBS) programs and early treatment with a restricted low-protein diet together with an adapted nutritional formula, and sometimes tetrahydrobiopterin (BH4), prevents severe neurological damage and allows a good quality of life and long-term survival [2]. However, despite early treatment, cognitive, psychiatric and brain alterations have also been reported [6–8].

White matter alterations associated with PKU are the most consistent neuroradiological finding. Indeed, neuropathological studies of untreated patients showed an altered myelination pattern. Magnetic resonance imaging (MRI) (T2 weighted images and FLAIR images) allows the identification of impaired myelination as high-signal intensity in periventricular white matter. In a review including 312 individuals with PKU aged between 0.9 and 49 years, Anderson and Leuzzi [3] reported abnormal white matter in 93% of cases viewed on T2; and some years later Mastrangelo et al., also in a mixed age cohort, reported alterations in white matter mostly seen during adulthood [9].

There is clear evidence that the degree of white matter (WM) abnormalities, and specifically diffusivity in WM, is influenced by metabolic control [10] and thus, it could change according to diet adherence and/or clinical evolution even in treated patients [3, 11] due to individual sensitivity to Phe [12]. Diffusion tensor imaging (DTI) is a suitable technique to identify and quantify regional white matter changes through diffusion [13], with the posterior-anterior decrement in mean diffusivity (MD) being the most consistently reported finding in PKU patient samples [13–23].

On the other hand, studies on volumetric gray matter subcortical structure in PKU are scarce. An early study published in 2005 performed manual volumetry of brain structures in a sample of 31 adult patients with PKU and found hippocampal reductions but no differences in the caudate, nucleus lentiformis, or thalamic structures [24]. In 2006, Pérez-Dueñas et al. [25] studied a sample including children and adolescents with PKU using the voxel-based morphometry (VBM) technique and reported increased volume of the ventral striatum. Using the same neuroimaging approach but in a sample including only adults, Pilotto et al. [26] reported significant gray matter reductions in the putamen and thalamus. Nevertheless, only putamen nuclei remained significant after correcting for multiple comparisons. In a small sample of 13 participants with PKU, mixing children, adolescents, and adults and performing manual volumetry of basal ganglia, Bodner et al. [27] reported increased volumes of putamen and a significant positive correlation between current Phe levels and putamen volume. A similar positive correlation was reported by Brown et al. [28] using automatic volumetry. To summarize, these discrepant results may be explained by methodological differences and/or the inclusion of dissimilar study groups, sometimes including patients with immature brains. Currently, only one study has focused on adult patients exclusively, and this observed reductions in the putamen volume [26].

Although traditionally it was considered uncommon, recent studies are increasingly demonstrating cortical gray matter involvement in the PKU setting. Pfaendner et al. [24] described global reductions of estimated gray matter volume in adult patients with PKU, and Pérez-Dueñas et al. [25] found gray matter reductions in the motor cortex and the left premotor cortex using the VBM approach. Later, Christ et al. [29] obtained average gray matter volumetric measurements for each major cortical lobe and reported a decrease in parietal cortex volume in the group with PKU (19 patients with an age range from 9 to 33 years) compared with the control group. Subsequent subregional analysis revealed significant differences in parietal (e.g., left precuneus, dorsal supramarginal gyrus) and occipital regions (e.g., left inferior occipital gyrus, right posterior collateral sulcus). Later, Muri et al. [30] reported whole brain mean cortical thickness reduction in adult patients with PKU based on residual scores obtained by considering intracranial volume and age. However, surface area on a whole-brain level did not differ between patients and controls. At the lobar level, patients showed a significantly thinner cortex in the left and right temporal, parietal, and occipital lobar regions of interest (ROIs). Conversely, Hawks et al. [19] found no significant group differences in cortical whole-brain measures (cortical gray matter volume, total surface area, and average cortical thickness). To the best of our knowledge, there are no previous studies that have demonstrated a vertex-wise cortical thickness reduction in adults with PKU.

In summary, current research suggests a link between elevated Phe levels, which may indicate Phe toxicity, and anomalies in brain anatomy [10] and neuropsychological functioning, mostly when referred to subcortical structures, even in treated patients with PKU [3, 10]. Nevertheless, most studies have been performed on children or on mixed child and adult samples. Only four structural MRI-quantified studies have included adults only [26, 28, 30, 31]. As adulthood is the ideal condition in which to study structural changes associated with PKU as no significant neurodevelopmental variations are expected, we selected a relatively large sample of adults with PKU under early dietary treatment in the current study. We primarily aimed to investigate differences in cortical and subcortical gray matter and global volumetric measures in comparison to healthy controls. As a secondary objective, we tested the possible relationship between structural alterations and metabolic and neuropsychological variables.

## Methods

### Participants

Participants in this study were recruited from the Adult Inherited Metabolic Disorders Unit at the Hospital Clínic (Barcelona, Catalonia, Spain). The total sample comprised 57 participants, 35 of whom were early-treated PKU or hyperphenylalaninemia (HPA) patients; the remaining 22 participants were healthy controls (HC) matched according to age, sex, and body mass index (BMI).

The inclusion criteria for PKU patients were: (1) age above 18 years old and (2) genetic diagnosis of PKU or HPA. The exclusion criteria for patients were: (1) intelligence quotient estimation below 70 according to Wechsler Adult Intelligence Scale 4th edition (WAIS-IV) tests, (2) pregnancy or planning a pregnancy during the study period, (3) active cancer, (4) severe chronic hepatic disease, (5) acute cardiovascular event in the 6 months prior to study inclusion, (6) common MRI contraindications, (7) claustrophobia, (8) pathological MRI findings other than mild white matter hyperintensities in long-TR sequences, and (9) MRI artifacts. The exclusion criteria for HC were the same as those applied to the PKU group.

This study was approved by the Bioethics Committee of the University of Barcelona (IRB00003099) and Hospital Clínic of Barcelona (HCB/2020/0552) and was conducted in accordance with the basic principles of the Declaration of Helsinki, among other relevant regulations and guidelines. All participants in this study provided signed written informed consent, after a complete explanation of the procedures involved.

### Clinical data

Sociodemographic information and clinical features of participants were also collected, including the date of PKU or HPA diagnosis, Phe monitoring using DBS, venous Phe, previous/current pharmacological treatments, use of adapted formulas (e.g., protein substitutes), consumption of dietetic supplements, subjective cognitive complaints through a clinical interview, and other medical diagnoses. Specific information regarding protein intake was also available, differentiating between natural protein intake and total (natural plus formula-derived protein) intake.

The Phe analysis was performed in DBS via tandem mass spectrometry (MS/MS) using the NeoBase™ 2 Non-derivatized kit (Revvity, Inc; Waltham, Massachusetts, U.S.). Briefly, to extract Phe from 3.2 mm of DBS, an organic compound solution that includes the deuterated Phe-d3 (internal standard) was added. Subsequently, 10 µL of this solution was directly injected into the MS/MS (Xevo-TQD; Waters Corp; Milford, Massachusetts, U.S.). Acquisition was performed in positive ionization and Multiple Reaction Monitoring modes using *Masslynx software* (Waters Corp). The run time was 2.5 min. The concentration of Phe was calculated based on the area relative to its internal standard, which has a known concentration, using *Neolynx software* (Waters Corp). The results are expressed in µmol/L.

Values of Phe obtained from DBS conformed to the index of dietary control (IDC), which was approximated using the median Phe levels measured in DBS recorded in the year prior to study inclusion (approximately 6–12 measurements per year for each patient) [30, 32].

### Neuropsychological assessment

The participants included in this study (both HC and patients with PKU) underwent a comprehensive neuropsychological assessment selected according to the most affected domains reported in previous literature [33, 34].The neuropsychological battery included (1) the Vocabulary subtest (WAIS-IV) used to estimate premorbid functioning, (2) the Similarities subtest (WAIS-IV), (3) the Arithmetic subtest (WAIS-IV), (4) the Digit Span subtest (WAIS-IV), which includes Digit Forward, Digit Backward and Digit Sequencing, (5) Letter-Number Sequencing (WAIS-IV), (6) Block Design (WAIS-IV), (7) Matrix Reasoning (WAIS-IV), (8) Digit-Symbol Coding (WAIS-IV), (9) Symbol Search (WAIS-IV), (10) Rey’s Auditory Verbal Learning Test (RAVLT), including immediate, delayed recall after 20 min, and recognition, (11) parts A and B of the Trail Making Test (TMT), (12) semantic (animals) and phonemic (letter “P”) fluency tests, and (13) the Rey-Osterrieth Complex Figure (ROCF), including copy and immediate recall. Behavior and executive functioning were also assessed through the self-reported Behavior Rating Inventory of Executive Function for Adults (BRIEF-A).

### MRI acquisition

High-resolution three-dimensional T1-weighted images were acquired in the sagittal plane (TR = 2400 ms, TE = 2.22 ms, TI = 1000 ms, flip angle 8°, 208 slices, FOV = 256 mm; 0.8 mm isotropic voxel) using a Siemens Magnetom Prisma 3 T scanner located at the Centre de Diagnostic per la Imatge of the Hospital Clínic de Barcelona (Catalonia, Spain). The scanning protocol used in this study also included T2-weighted images in axial orientation (TR = 3200 ms, TE = 563 ms; 512x307 matrix, flip angle 120°, slice thickness 0.8 mm with a 1.5 mm interslice gap) and an axial FLAIR sequence (TR = 9000 ms, TE = 125 ms; TI = 2500; 250×171 matrix, flip angle 150°, slice thickness 4 mm).

### Cortical thickness and volumetric measures

Cortical thickness was estimated using the automated *FreeSurfer* stream version 7.1 available at https://surfer.nmr.mgh.harvard.edu/. The procedures in *FreeSurfer* included removal of non-brain data, registration to Talairach space, intensity normalization, and tessellation of the gray matter and white matter boundaries, automated topology correction, and accurate surface deformation following intensity gradients to identify tissue borders. Cortical thickness was calculated as the distance between the white and gray matter surfaces at each vertex of the reconstructed cortical mantle (https://freesurfer.net/fswiki/FreeSurferMethodsCitation). Results for each subject were visually inspected, and the appropriate manual corrections were performed to ensure the accuracy of registration, skull stripping, segmentation, and cortical surface reconstruction (https://surfer.nmr.mgh.harvard.edu/fswiki/FsTutorial/TroubleshootingData).

Deep gray matter mean volumes (e.g., in the thalamus, putamen, pallidum, caudate, hippocampus, amygdala, accumbens, and brainstem) and total cortical and subcortical gray matter were extracted through *FreeSurfer* version 7.1 [35].

The volumetric measures are presented in ratios, dividing the brain structure volume by the estimated total intracranial volume (ICV) and multiplying the result by 100.

### Statistical analyses

Analyses of sociodemographic, neuropsychological, volumetric, and clinical data were performed using IBM SPSS Statistics 27.0.1.0. Continuous variables were analyzed using Student t-tests or Mann–Whitney U tests to accommodate both normal and non-normal distributions, while categorical variables were assessed through Pearson’s chi-squared test. The Mann–Whitney U test specifically addressed non-normal variables. However, significance in neuropsychological and volume data was reported exclusively for values that passed multiple comparison corrections that had survived family-wise error rate (FWE) correction and the false discovery rate (FDR) through MATLAB (v.R2020b). In all cases the significance threshold was set at a *p*-value < 0.05.

The study also explored relationships between volume variables, neuropsychological performance and metabolic control assessed by IDC, using pairwise Spearman’s rank correlation analyses, with a significance threshold of *p* < 0.05.

Intergroup cortical thickness analyses were conducted through a vertex-by-vertex general linear model (GLM) with *FreeSurfer* version 7.1, including cortical thickness as a dependent factor and group as an independent factor. Cluster-extent correction for multiple comparisons was tested using the Monte-Carlo simulation with 10,000 iterations implemented in *FreeSurfer*, in order to prevent false positives. Only the clusters that survived FDR with the statistical significance threshold set at *p* < 0.05 were reported.

The “ggseg” package in R was used for the graphical visualization of volumetric data.

## Results

The sociodemographic and clinical characteristics of the participants are summarized in Table 1. There were no between-group differences in age (U = 311.500; *p* = 0.228), sex (X^2^ = 0.127; *p* = 0.722), parents’ education (U = 382.000; *p* = 0.961), or BMI (t = 0.889; *p* = 0.189), but there were differences in years of education (U = 218.500; *p* = 0.006), psychiatric comorbidities (X^2^ = 8.568; *p* = 0.003) and natural protein intake (U = 60.000; *p* =  < 0.001).

**Table 1:** Sociodemographic and clinical characteristics of the participants (HC vs PKU)

	**HC** *n* = 22	**PKU/HPA** *n* = 35	**Statistics**	***p-value***
Age, years	34.73 ± 8.87 (22–56)	31.86 ± 8.30 (18–55)	311.500^U^	0.228
Sex, male (%)	20 (38.46%)	14 (41.18%)	0.127^X^^2^	0.722
Education, years	17.95 ± 3.36 (9–23)	15.29 ± 3.359 (10–24)	218.500^U^	0.006^*^
Parents education, years	10.75 ± 4.03 (4–18)	10.40 ± 3.66 (3–19)	382.000^U^	0.961
BMI	24.54 ± 3.75 (18.95–33.74)	23.72 ± 3.17 (18.25–30.38)	0.889^t^	0.189
IDC-Phe levels (µmol/L)	-	519.09 ± 248.48 (109–1000)	-	-
Good control^a^, yes (%)	-	20 (57.14%)	-	-
Synthetic BH4^b^, yes (%)	-	9 (28.12%)	-	-
Classical PKU^c^				
Early dx, yes (%)	-	27 (77.14%)	-	-
Late dx, yes (%)	-	3 (8.57%)	-	-
HPA, yes (%)	-	5 (14.28%)	-	-
Protein intake				
Natural protein	76 ± 20.80	32.29 ± 21.57	60.000^U^	< 0.001^*^
Total protein^d^	76.79 ± 20.49	89.04 ± 23.92	254.000^U^	0.061
Psychiatric comorbidities, yes (%)	0 (0%)	11 (47.83%)	8.568^X^^2^	0.003^*^
Subjective cognitive complaints, yes (%)	14 (63.64%)	23 (71.87%)	0.026^X^^2^	0.873

Values denote mean ± SD (range) or numbers (frequencies)

Statistical tests used: ^X^^2^Chi-Squared, ^t^t-test, ^U^Mann-Whitney

*Abbreviations*: *BMI* body mass index, *BRIEF-A* Behavior Rating Inventory of Executive Function for Adults (Self-Report form), *dx* diagnosed, *HC* healthy controls, *IDC* Index Dietary Control, *PKU/HPA* patients with phenylketonuria/hyperphenylalaninemia, *WAIS-IV* Wechsler Adult Intelligence Scale-IV

^*^Statistical significance highlight, threshold set at *p* < 0.05

^a^According to European guidelines standards (Phe values < 600 μmol/L) [36]

^b^Sapropterin dihydrochloride (Kuvan®, *BioMarin Pharmaceutical Inc*.; Novato, California, U.S.), a synthetic form of the tetrahydrobiopterin (BH4), a cofactor for phenylalanine hydroxylase gene

^c^Classical PKU is divided into ‘early diagnosed’ (< 3 months of age) and ‘late diagnosed’ (≥ 3 months-< 7 years) [37]. Within the group of early diagnosed 26 of them were at New-born screening and one of them at 2 months of age. Late diagnosed PKU were diagnosed at the age of 3 years old

^d^Total protein refers to natural plus formula-derived protein

Adult patients with PKU/HPA showed significantly lower performance than HC in the Verbal Comprehension Index (VCI) (*t* = 2.08; *p* = 0.043) and prorated Full Scale IQ (FSIQ) (*t* = 2.67; *p* = 0.010) from the WAIS-IV (Table 2). Only FSIQ survived FDR correction (F = 7.107; *p* = 0.035).

**Table 2:** Neuropsychological performance of the participants

	**HC** *n* = 22	**PKU/HPA** *n* = 35	**statistics**	***p-value***	***pFDR***
Vocabulary	38.27 ± 5.650	34.66 ± 7.447^a^	246.500^U^	0.063	0.090
Similarities	25.05 ± 3.848	23.19 ± 3.831^a^	248.500^U^	0.067	0.104
VCI	116.14 ± 15.050	108.06 ± 13.320^a^	2.076^t^	0.043^*^	0.090
Block design	44.64 ± 8.295	44.66 ± 11.545	-0.007^t^	0.994	0.996
Matrix reasoning	21.23 ± 2.468	19.34 ± 4.051	286.500^U^	0.103	0.346
PRI	102.45 ± 12.439	96.89 ± 13.503	1.562^t^	0.124	0.422
Digit	25.14 ± 4.804	24.74 ± 4.415	384.000^U^	0.987	0.926
*Forward*	8.64 ± 2.128	8.49 ± 1.652	385.000^U^	1.000	0.926
*Backwards*	8.95 ± 2.149	8.74 ± 1.853	366.000^U^	0.752	0.926
*Sequencing*	7.55 ± 1.471	7.51 ± 1.755	380.500^U^	0.940	0.996
Arithmetic	13.18 ± 2.373	11.81 ± 3.267^a^	261.000^U^	0.107	0.120
Letter-number sequencing	19.27 ± 2.529	18.54 ± 2.267	295.500^U^	0.139	0.480
WMI	95.32 ± 11.721	89.63 ± 14.334	299.500^U^	0.159	0.442
Symbol search	33.23 ± 5.589	32.34 ± 7.662	0.503^t^	0.617	0.909
Digit-symbol coding	78.27 ± 12.456	70.74 ± 14.650	1.998^t^	0.051	0.346
PSI	104.77 ± 11.351	98.17 ± 11.610	271.500^U^	0.061	0.346
Fluency tests					
*Semantic (animals)*	24.77 ± 3.963	22.68 ± 5.470^b^	270.500^U^	0.082	0.179
*Phonetic (letter P)*	16.05 ± 4.134	14.68 ± 3.470^b^	311.500^U^	0.292	0.179
TMT					
*Part A*	28.68 ± 8.855	31.89 ± 9.588	312.000^U^	0.321	0.480
*Part B*	68.50 ± 25.194	70.69 ± 20.786	360.000^U^	0.682	0.926
FSIQ	105.95 ± 12.167	97.34 ± 11.307^a^	2.666^t^	0.010^*^	0.035^*^
RAVLT					
*Total*	54.09 ± 6.575	51.00 ± 8.363	312.500^U^	0.234	0.422
*Recall*	11.77 ± 1.688	11.06 ± 2.555	323.000^U^	0.305	0.480
ROCF					
*Copy*	32.909 ± 2.580	32.771 ± 3.828	368.500^U^	0.231	0.926
*Immediate recall*	21.818 ± 6.563	21.471 ± 5.326	0.219^t^	0.828	0.946
BRIEF	109.88 ± 17.885	107.21 ± 20.898	917.000^U^	0.577	0.577
BRIEF, altered (%)	3 (13.636%)	3 (8.571%)	0.368^X^^2^	0.544	0.577

Values denote the mean ± SD of raw scores and index of the WAIS-IV

Statistical tests used: ^X^^2^Chi-Squared, ^t^t-test, ^U^Mann-Whitney, FDR correction

*Abbreviations*: *BRIEF* Behavior Rating Inventory of Executive Function for Adults, *FSIQ* Full Scale IQ, *HC* healthy controls, *PKU/HPA* patients with phenylketonuria/hyperphenylalaninemia, *PRI* Perceptual Reasoning Index, *PSI* Processing Speed Index, *RAVLT* Rey Auditory Verbal Learning Test, *ROCF* Rey-Osterrieth Complex Figure, *TMT* Trail Making Test, *VCI* Verbal Comprehension Index, *WMI* Working Memory Index

^*^Statistical significance highlight, threshold set at *p* < 0.05

^a^*n* = 32

^b^*n* = 34

**Fig. 1:**
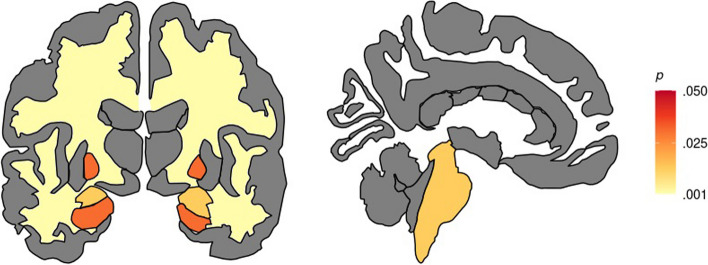
PKU showed statistically significant lower volumes, presented as ratio scores (volume/intracranial volume*100), in the pallidum (U = 224.000; *p* = .008), hippocampus (U = 243.000; *p* = .020), amygdala (U = 200.000; *p* = .002), brainstem (t = 3.17; *p* = .006) and total cerebral white matter (U = 175.000; *p* = .001), in comparison with HC

The PKU group showed significantly lower volumes than HC in the pallidum (U = 224.000; *p* = 0.008), hippocampus (U = 243.000; *p* = 0.020), amygdala (U = 200.000; *p* = 0.002), brainstem (t = 3.17; *p* = 0.006) and total cerebral white matter (U = 175.000; *p* = 0.001) (Table 3; Fig. 1). These results remained significant after adjustment for age.

**Table 3:** Subcortical and global volumetric ratios of the participants

	**HC** *n* = 22	**PKU/HPA** *n* = 35	**Statistics**	***p-value***	***pFDR***
Thalamus	0.532 ± 0.056	0.519 ± 0.045	340.000^U^	0.461	0,421
Caudate	0.245 ± 0.030	0.237 ± 0.023	351.000^U^	0.577	0,357
Putamen	0.343 ± 0.039	0.332 ± 0.028	1.239^t^	0.221	0,327
Pallidum	0.142 ± 0.014	0.133 ± 0.012	224.000^U^	0.008^*^	0,031^*^
Hippocampus	0.293 ± 0.034	0.273 ± 0.023	243.000^U^	0.020^*^	0,031^*^
Amygdala	0.116 ± 0.012	0.108 ± 0.009	200.000^U^	0.002^*^	0,013^*^
Accumbens	0.034 ± 0.006	0.033 ± 0.004	0.656^t^	0.515	0,508
Brainstem	1.510 ± 0.172	1.390 ± 0.116	3.172^t^	0.006^*^	0,013^*^
Cortical gray matter	33.363 ± 3.195	32.097 ± 2.166	289.000^U^	0.116	0,685
Subcortical gray matter	4.069 ± 0.379	3.885 ± 0.236	294.000^U^	0.136	0,056
Cerebral white matter	15.883 ± 1.565	14.676 ± 1.129	175.000^U^	0.001^*^	0,013^*^
Mean cortical thickness	2.521 ± 0.080	2.535 ± 0.068	-0.723^t^	0.236	0,507
Total estimated intracranial volume (cm^3^)	1443.684 ± 198.753	1509.057 ± 187.899	-1.251^t^	0.216	0,327

Values denote the mean ± SD of volumetric ratio scores

Statistical tests used: ^t^t-test, ^U^Mann-Whitney, FDR correction

*Abbreviations*: *HC* healthy controls, *PKU/HPA* patients with phenylketonuria/hyperphenylalaninemia

^*^Statistical significance highlight, treshold set at p<0.05

**Fig. 2:**
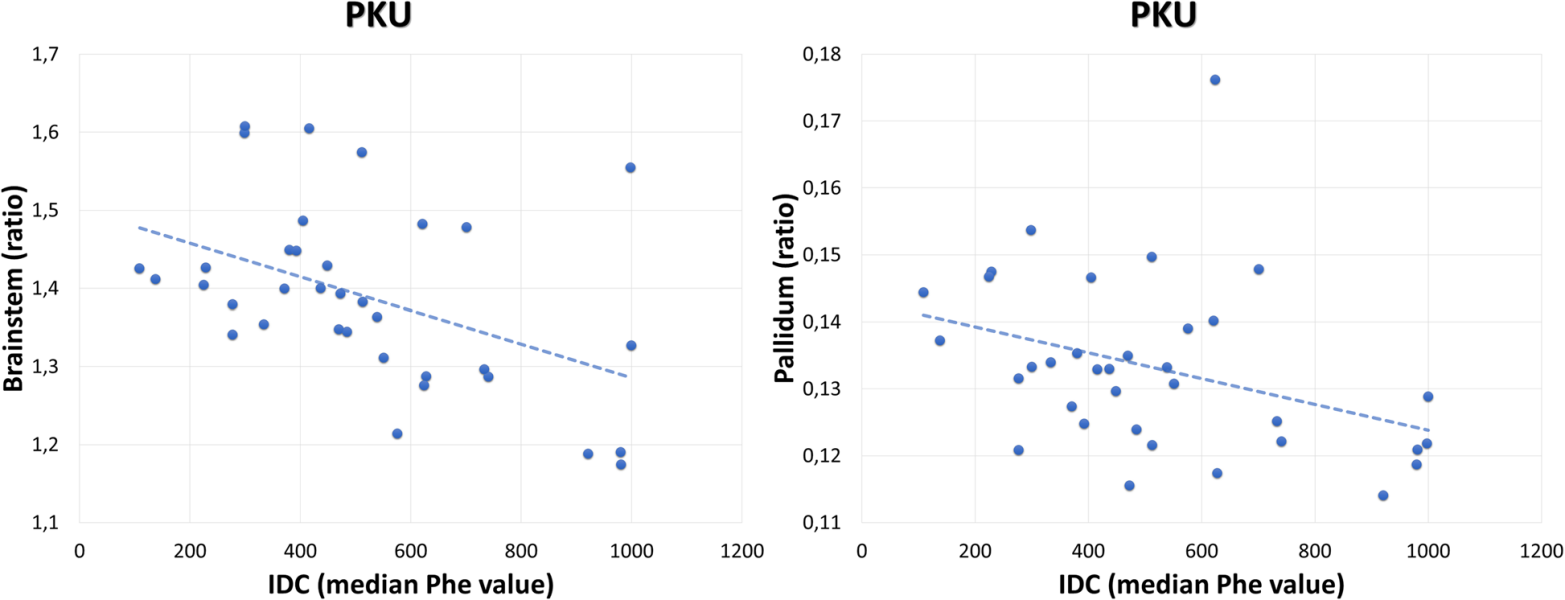
Significant negative correlations between brainstem and IDC (*r* = -0.455, *p* = .006) and pallidum and blood IDC (*r* = -0.417; *p* = .013) in the PKU/HPA group

IDC in PKU patients was negatively correlated with the pallidum (*r* = -0.417; *p* = 0.013) and brainstem (*r* = -0.455, *p* = 0.006) volumes (Fig. 2). No correlations were found between volume and neuropsychological performance or natural protein intake.

## Discussion

We found gray matter reductions in subcortical gray matter in PKU. Specifically, patients differed from controls in the volumes of pallidum, hippocampus, amygdala, and brainstem. In addition, whereas total cerebral white matter volume was decreased, no changes were detected in the volumetric or thickness measures of cortical gray matter in early treated adult PKU.

Basal ganglia abnormalities have previously been reported in both directions i.e. increased [25, 27] and decreased [31] volumes. Since volumetric increases have only been seen in samples including children and adolescents, it can be argued that the effects of Phe in basal ganglia may depend on the degree of maturation and/or the long-term effects of Phe levels. Our results on basal ganglia are consistent with the only previous study performed in adults with PKU [31]. However, a discrepancy with our observations should be highlighted. Whereas Pilotto et al. [31] reported significant differences in the putamen, we found significant results in the pallidum. Both basal ganglia structures have a high density of dopamine receptors, as does the brainstem, which was also reduced in volume in our sample. In patients with PKU, intracerebral dopamine depletion has been postulated due to reduced Tyr uptake and altered dopamine synthesis [38]. Therefore, we speculate that Phe toxicity could be associated with specific abnormalities in dopaminergic structures. In this regard, brainstem reduction has also been described in neuropathological studies of non-treated patients with PKU [39]. Our study provides the first evidence on brainstem volume reduction in treated PKU patients using an automatic MRI segmentation procedure.

Moreover, we also found volumetric decreases in limbic structures i.e. the hippocampus and amygdala. Hippocampal reduction in adult PKU patients was reported by Pfaendner et al. [24] using a manual volumetry approach. They found a 14.5% reduction in patients compared to controls. Wesonga et al. [20] found that MD values of the hippocampus differed between patients with PKU and controls, and also reported increased values with aging in PKU individuals but not in controls. The age by group interaction was also significant. Similar results were reported by Hawks et al. [19] Thus, hippocampal involvement in PKU seems to be a consistent finding. Interestingly, we also found amygdala reductions that could be related to the emotional changes seen in some patients. This structure was not included in the ROI analyses [22, 24, 27]. Our results point to the need to pay special attention to this structure in further multicenter studies.

Moreover, we found no significant differences in whole brain gray matter volume, global cortical thickness, or cortical thickness maps. Whereas these results are consistent with the findings of Hawks et al. [19], who applied a similar MRI approach to our group, they conflict with recent results from Muri et al. [30], who suggested that cortical thickness is a particularly sensitive marker for gray matter alterations. It is noteworthy that previous results on cortical thickness were based on mean cortical thickness measures using global or ROI measurements. The ROI approach reduces problems related to multiple comparisons and is advantageous when an a priori hypothesis is clearly stated but not when the objective is the study of whole-brain cortical involvement. In this regard, no whole-brain vertex-wise approaches had been reported before the current study. Previous research has even identified variations in the gray matter of the cerebellum [40]; however, this region was not examined in our study.

Finally, our correlation analyses showed that IDC was negatively correlated with pallidum shrinkage. Of note, this correlation was congruent with the results obtained in group comparisons showing reductions in this subcortical structure. In our sample, patients with PKU presented a reduced brainstem volume, and this reduction was related to higher levels of Phe.

The strengths of our study lie in the inclusion of a relatively large sample of early-treated adults with PKU/HPA and well-matched HC, along with a detailed and comprehensive neuropsychological examination. Furthermore, we implemented a neuroimaging approach including both gray and white matter volumetric metrics, but also mean global measures and a surface-based approach to study whole-brain vertex-wise cortical changes. However, several limitations should also be acknowledged. First, although our cohort was more homogeneous (adults) compared to previous studies in the field, 26 individuals were diagnosed with NBS, and four were diagnosed between 2 months and 3 years old, so dietary treatment was initiated later for them. However, as patients with an estimated intelligence quotient < 70 (WAIS-IV) were not included, it is unlikely that our results were subjected to significant bias due to sample variability. Second, while classically the IDC is calculated as the median of Phe levels and the mean of all medians throughout the period of interest (childhood, youth, adulthood) [32], we focused on the median of Phe-DBS levels in the year prior to study inclusion. The average lifetime Phe levels for participants were not quantitatively available. This limitation arose because while pediatric history records were accessible for 18 participants, data were not available for all participants. As the calculated 1-year IDC allows the standardization of the same measure for the whole group and correction for sporadic decompensations (e.g., illness, dietary abandon), the selected approach offers a good picture of adults’ metabolic control. Finally, our study did not include an exhaustive assessment of executive functions such as the n-back task [17]. The executive functions in our study were assessed using the fluency test and the Trail Making test. In addition we used The BRIEF as a screening tool, but this is not able to detect the clinical phenotype of those individuals showing mild executive dysfunction or to detect the correlates of basal ganglia volumetric reductions. In this regard, previous studies reported consistent differences between groups in certain executive domains [34], and new toolboxes, which also integrate cognitive indices and additional motor evaluations, have recently been introduced for this purpose [41].

The incorporation of MRI and neuropsychological assessments is important in the initial evaluation and subsequent monitoring of PKU patients. These diagnostic tools may enhance our understanding of how metabolic control influences neuropsychological outcomes, to achieve individualized care strategies. Therefore, future research with multicenter cohorts is crucial, as it will enable the development of predictive models that can guide personalized treatment approaches more effectively.

In conclusion, this study expands on previous findings showing global white matter reduction but also gray matter abnormalities in adult patients with PKU. Early-treated PKU mainly gives rise to volumetric reductions in basal ganglia, brainstem, and limbic subcortical structures that are related to poorer metabolic control, without evidence of neocortical involvement.

## Data Availability

The data that support the findings of this study are available from the corresponding author upon reasonable request.

## Abbreviations

BH4: Tetrahydrobiopterin
BMI: Body mass index
BRIEF-A: Behavior Rating Inventory of Executive Function for Adults
DBS: Dried bloodspot
DTI: Diffusion tensor imaging
FEW: Family-wise error rate
FDR: False discovery rate
FSIQ: Full Scale IQ
GLM: General linear model
HC: Healthy controls
HPA: Hyperphenylalaninemia
ICV: Intracranial volume
IDC: Index of dietary control
MD: Mean diffusivity
MRI: Magnetic resonance imaging
MS/MS: Mass spectrometry
NBS: Newborn screening
Phe: Phenylalanine
PKU: Phenylketonuria
VBM: Voxel-based morphometry
VCI: Verbal Comprehension Index
WAIS-IV: Wechsler Adult Intelligence Scale 4th edition
WM: Withe matter
